# H_2_S and Oxytocin Systems in Early Life Stress and Cardiovascular Disease

**DOI:** 10.3390/jcm10163484

**Published:** 2021-08-06

**Authors:** Oscar McCook, Nicole Denoix, Peter Radermacher, Christiane Waller, Tamara Merz

**Affiliations:** 1 Institute for Anesthesiological Pathophysiology and Process Engineering, Ulm University Medical Center, 89081 Ulm, Germany; nicole.denoix@uni-ulm.de (N.D.); peter.radermacher@uni-ulm.de (P.R.); tamara.merz@uni-ulm.de (T.M.); 2 Clinic for Psychosomatic Medicine and Psychotherapy, Ulm University Medical Center, 89081 Ulm, Germany; 3 Department of Psychosomatic Medicine and Psychotherapy, Nuremberg General Hospital, Paracelsus Medical University, 90471 Nuremberg, Germany; Christiane.Waller@klinikum-nuernberg.de

**Keywords:** hydrogen sulfide, oxytocin, oxytocin receptor, cardiovascular disease, early life stress, cystathionine-γ-lyase, cystathionine β-synthase, 3-mercaptopyruvate sulphurtransferase

## Abstract

Today it is well established that early life stress leads to cardiovascular programming that manifests in cardiovascular disease, but the mechanisms by which this occurs, are not fully understood. This perspective review examines the relevant literature that implicates the dysregulation of the gasomediator hydrogen sulfide and the neuroendocrine oxytocin systems in heart disease and their putative mechanistic role in the early life stress developmental origins of cardiovascular disease. Furthermore, interesting hints towards the mutual interaction of the hydrogen sulfide and OT systems are identified, especially with regards to the connection between the central nervous and the cardiovascular system, which support the role of the vagus nerve as a communication link between the brain and the heart in stress-mediated cardiovascular disease.

## 1. Early Life Stress: Definition and Cause

The significant role that early life stress (ELS) (e.g., poverty, childhood maltreatment such as physical, sexual, and psychological abuse, maternal separation and/or neglect (CM), and psychological comorbidities) plays in the development of cardio-metabolic disease has recently gained prominence [1]. Basu et al. reviewed the incidence of child maltreatment in association with cardiovascular disease (CVD) (e.g., coronary artery disease, myocardial infarction (MI), stroke, ischemic heart disease) and found a positive association of 91.7% and for hypertension 61.5% [2]. Furthermore, it has recently been established that ELS can contribute to an increased risk of mortality and different CVDs: arthrosclerosis, MI, stroke, arterial hypertension, chronic heart failure, and ischemic and coronary heart disease [1,3]. In a very recent population-based retrospective cohort study in the United Kingdom, Chandan et al. report that child maltreatment leads to an increased risk for CVD and hypertension, a doubling of type 2 diabetes, and of all-cause mortality [4].

Recently the gasotransmitter hydrogen sulfide (H_2_S) and the neuroendocrine oxytocin (OT) systems have been shown to interact and play parallel roles in the heart and brain in response to trauma, both physical and psychological [5,6,7,8,9,10]. Trauma can result from either a physical injury or be of psychological origin, the latter being trauma instigated by a deep emotional pain that threatens the integrity of the self [11]. Psychological trauma is characterized by an intense emotional response to a perceived life-threatening situation and inability to cope. Childhood trauma is defined as “physical/sexual abuse, medical trauma, motor vehicle accident, acts of terrorism, war experiences, natural and human-made disasters, witnessed homicides/suicides” [11]. Physical trauma is normally associated with an impact against the body and/or physical injury. ELS and childhood trauma have an extremely high incidence with 30–40% of the general adult population reporting having experienced some kind of early life adversity [11]. Recent research has established that both physical and psychological trauma share physiological correlates [12,13]. These include the OT and H_2_S systems which are reported to be cardio-protective and display antioxidant and anti-inflammatory properties in models of psychological and physical trauma [12,14,15,16,17]. The purpose of this perspective is to explore the role of the H_2_S and OT systems in the cardiovascular system and their mediating potential in ELS. For the purposes of clarity and simplification, the term ELS will be used from now on to include early life adversity, CM, and childhood trauma.

## 2. H_2_S System

H_2_S is classified as a gasotransmitter along with the previously characterized nitric oxide (NO) and carbon monoxide (CO). It is produced endogenously and has reportedly pleiotropic effects in mammalian cells and tissues [18]. H_2_S endogenous production is threefold: (1) it can be enzymatically produced by cystathionine γ-lyase (CSE), cystathionine β-synthase (CBS), and 3-mercaptopyruvate sulphurtransferase (3MST) [19]; (2) non-enzymatic pathways that generate H_2_S include thiosulphate, glucose, polysulfides, glutathione, and elemental sulfur; (3) the gut microbiota is another source of H_2_S, almost half of the fecal H_2_S is bacterially derived, and sulfate-reducing bacteria release H_2_S as they oxidize organic compounds [20].

All of the above-mentioned pathways, except for the bacterial microbiota, are found in the heart and vasculature. In particular, CSE and 3MST have been reported to be expressed in the vasculature and hence play a role in the regulation of smooth muscle vasomotor tone. CBS expression has been shown to be inversely correlated with homocysteine levels, which is a factor in the progression of CVD [21]. There are a number of reviews on H_2_S and its protective effects in the cardiovascular system [17,19,20,21,22,23,24,25,26,27,28,29]. The expression of H_2_S-producing enzymes has been identified in the cardiovascular system, specifically in the following cell types: smooth muscle cells, cardiomyocytes, endothelial cells and immune cells [9,10,12,19,30,31].

H_2_S has been shown to play a role in modulating the cardiovascular system as a basal vasorelaxant, a blood pressure and heart rate regulator [32,33], and by inducing angiogenesis [19,34] through hypothalamic control. The results of animal (see Table 1) and human studies (see Table 2) investigating the regulation of H_2_S in the cardiovascular system are summarized below. In humans, H_2_S levels were correlated with disease severity in hypertensive patients [35] and are significantly reduced in hypertensive children (see Table 2) [36]. Albeit, in general, reported H_2_S levels must be taken with some degree of skepticism in that there is no agreed upon standard for H_2_S measurements, and the discrepancy in the literature regarding blood sulfide concentrations is considerable, varying by up to three orders of magnitude as reviewed by McCook et al. [37,38]. In rodents, the administration of H_2_S improved myocardial fibrosis, reduced oxidative stress and hypertension (see Table 1) [24]. The administration of sodium hydrosulfide (NaHS), an H_2_S releasing salt, in a rat model of hemorrhagic shock significantly reduced metabolic acidosis while simultaneously attenuated inducible nitric oxide synthase (iNOS) expression and NO production in the heart and aorta (see Table 1) [39]. The H_2_S-dependent vasoactive effects are mediated by downstream signaling cascades that stimulate Akt-dependent endothelial nitric oxide synthase (eNOS). Interestingly, the H_2_S and OT systems share these downstream signaling mechanisms which converge on the same nitric oxide synthase (NOS)/NO-dependent pathway [12,17,40].

## 3. Oxytocin/Oxytocin Receptor

The neuroendocrine OT system, in contrast to the highly diffusible gaseous mediator H_2_S, that does not require a membrane receptor, is based on a ligand–receptor interaction. Thus, the nonapeptide OT requires the presence of the OT receptor (OTR), a member of the G-protein coupled receptor family Gq for its mode of action. The binding of OT to its receptor stimulates pro-survival kinases such as ERK and PI3K/Akt, which can in turn activate eNOS or CSE (H_2_S) [59]. The NO-mediated vasodilatory effects of OT are at least in part also reported to regulate blood pressure [60,61] and body fluid homeostasis, through an interaction with H_2_S [14,62]. OTR expression has been detected in cardiomyocytes, vasculature (smooth muscle cells and endothelium), macrophages, peripheral blood mononuclear cells and cardiac fibroblasts [9,12,63,64,65,66,67,68]. There are a number of recent reviews available on the role of OT in the heart [60,63,69,70] which interestingly reflect the fact that OT shares many of the properties also reported for H_2_S, e.g., increase of glucose uptake in cardiac cells, anti-inflammatory and antioxidant activity [71,72], blood pressure lowering capacities via NO-mediated vasodilation [73], negative inotropic and chronotropic effects, natriuretic effects, and effects on endothelial cell growth [60,74,75,76].

The same discrepancy as for the reported H_2_S values also holds for OT measurements: there are major difficulties in the detection of OT, and reported concentrations are not very reliable, ranging from 1–1000 pg/mL in humans as reviewed by Szeto et al. [68,77]. It is also known to be unstable due to its short half-life (3–5 min) [68]. OT and arginine-vasopressin (AVP) share high sequence and structural homology. Thus, OT can also act through the AVP receptor (AVPR) system [78] and vice versa due to its 57% receptor homology and the fact that at the ligand level, they only differ in two amino acids (3 and 8). AVP can bind to the OTR with the same affinity that it binds to AVPRs, reviewed by Stoop et al. and Dumais et al. [78,79,80,81]. OT administration reveals dose-dependent reductions in body temperature and heart rate. These effects were proposed to be mediated through the AVPR1a [82]. Although they share a close homology, the AVPR1a is less affected by gonadal hormones than OTR [80]. The crosstalk amongst the receptors have led to much speculation ranging from OT and AVP having similar to diametrically opposite effects. Given the fact that OT can activate AVPR and, reciprocally, the AVP ligand can activate the OTR, it is more likely that it is the receptor activation and its specific location that may mediate the effects rather than the ligand itself [81]. To date, there are associations but there is no knowledge of what this reciprocal binding and interaction exactly elicit; a thorough discussion is beyond the scope of this work and has been recently reviewed [80,83,84]. In the following section, a brief summary of the cardiovascular interaction of the endogenous H_2_S and OT systems will be provided (for a more thorough review, see Denoix et al. [12]).

## 4. H_2_S and Oxytocin in Cardiovascular Disease

One of the problems encountered in delineating the interaction of the H_2_S and OT systems in CVD and trauma is the fact that there is an imbalance of the literature and research currently available: reports on OT in psychological trauma and H_2_S in physical trauma are abundant, whereas the inverse, H_2_S in psychological trauma and OT in physical trauma, are more limited [12]. In the following sections, a review of the literature for the H_2_S and OT systems in trauma, both physical and psychological, as they pertain to CVD will be addressed.

Atherosclerosis, i.e., the formation of fibro-fatty lesions in the vascular wall is the main cause of death from CVD [85], is characterized by increased low density lipoproteins, attenuated high density lipoproteins, oxidative stress, endothelial dysfunction, reduced NO bioavailability and inflammation [85,86]. Interestingly, chronic cardiovascular pathology has been associated with dysfunctional release of endogenous H_2_S (see Table 1 and Table 2) [19]. Both H_2_S [41,87,88] and OT [77,89] have been reported to reduce atherosclerotic plaque formation and mitigate inflammation after exogenous administration.

In an effort to design more translationally relevant pre-clinical trials, experiments have been performed with a pig strain, Familial Hypocholesteremia Bretoncelles Meishan (FBM), displaying human-like coronary atherosclerosis [90]. The FBM is a crossbreed of the Rapacz pigs, characterized by a homozygous R84C low-density lipoprotein (LDL) receptor mutation associated with recessive Familial hypercholesterolemia in pigs [91] which develop marked atherosclerosis and consecutive CAD upon an atherogenic diet [90]. FBM swine exhibit significantly elevated cholesterol levels, increased levels of isoprostanes, decreased levels of plasma nitrite/nitrate, well-established markers of lipid peroxidation, and NO availability, as well as significantly lower creatinine clearance compared to healthy German landrace swine [31]. In recent studies on resuscitated, co-morbid, septic FBM pigs, CSE, arguably the most important source of endogenous H_2_S in the cardiovascular system, displayed significantly reduced tissue expression in the coronary arteries [30], kidney [92], and myocardium [31]. These were associated with increased troponin levels, reduced cardiac OTR expression [10] and a lower cardiac output [30]. Interestingly, in their naïve state without septic shock, the FBM pig strain already presented with decreased CSE expression in the media of the coronary artery and elevated nitrotyrosine formation a marker of nitrosative and oxidative stress (see Table 1) [30].

Overall, these observations agree well with the fact that atherosclerosis and hypertension are associated with reduced levels of CSE [41]. Wang et al. [59] propose that since both H_2_S and OT are able to act via regulation of NO that CSE may be able to mediate cardio-protection by upregulating OTR through the reperfusion injury salvage kinase (RISK) pathway [5,59]. The RISK pathway has been suggested to be the downstream molecular pathway, where H_2_S and OT signaling converge in cardioprotection in atherosclerosis [59]. The RISK pathway is activated in endothelial cells, through the activation of eNOS/NO as an angiogenic and vasodilating factor. In other cells, such as cardiomyocytes, RISK-activated pathways regulate apoptosis and antioxidant signaling [12]. RISK activation leads to PI3K Akt, eNOS cascades, and ERK 1/2 activation [59], which in turn promotes reperfusion by stimulating cell migration and angiogenesis. The PI3K/Akt cascades are also activated through H_2_S and are reported to promote myocardial protection [93]. Supporting the above claims, Kobayashi et al. [42] reported that post-infarct administration of OT significantly attenuated MI size, left ventricular (LV) function, and remodeling by both activating and upregulating the OTR, which led to stimulating the pro-survival signals Akt, ERK, and STAT3. The authors suggest that these pro-survival signals contributed to the cardio protective effects [42]. Furthermore, they also reported increased levels of phosphorylated p-eNOS and suggested that the Akt-eNOS signaling played a role in the beneficial effects of OT in their model (see Table 1) [42]. So far, these signaling pathways for H_2_S- and OT-mediated myocardial protection have only been identified in animal models [59,93]. Nonetheless, evidence in humans points to the fact that heart failure patients, with severe end-stage cardiomyopathy and reduced heart function, presented with significantly lower H_2_S levels in contrast to age-matched controls [17], which was associated with reduced NO levels [56]. These observations led to a phase I clinical trial on an H_2_S prodrug SG1002 in healthy and heart failure patients with promising results. The data suggest that SG1002 was well tolerated and increased H_2_S blood levels and NO bioavailability (see Table 2) [56]. 

The role of OT in myocardial injury has also been evaluated in a pig model of MI revealing a rather complex interaction: in animals treated immediately after the MI for up to seven days, OT had significantly decreased fraction shortening and had no effect on lesion size; one animal even died from a fatal ventricular arrhythmia [43]. At 8 days post MI, pigs with high basal endogenous OT levels receiving OT treatment displayed deterioration of ventricular function and increased infarct size at 28 d post MI in comparison to placebo animals with high endogenous OT levels. Thus, OT administration led to a significant adverse effect in animals with high endogenous OT levels [43]. In contrast, the low endogenous OT group that received exogenous OT administration starting at day 8 post MI had reduced infarct size [43]. Interestingly, the authors reported a trend towards increased infarct size in the low endogenous OT placebo group compared to the high endogenous placebo arm. Furthermore, administration of OT reduced cardiac OTR expression in high endogenous OT treated animals, but not in low endogenous OT treated ones [43]. Rightly, the authors point out that there is very limited information on the cardiac OTR expression, and their report was the first to show a decrease in cardiac OTR protein after a long-term infusion of OT (see Table 1) [43]. Unfortunately, no basal OTR levels, neither in the naïve state nor post MI, were provided in these experiments. Thus, there is no way of assessing the receptor–ligand interactions in the heart in the uninjured state, nor how the expression of the OTR is modulated by injury. It is clear, though, that the high endogenous levels of OT where protective but were deleterious in combination with exogenous OT administration.

Intriguingly, the authors stratified their pigs into low (<115 pg/mL) and high (>115 pg/mL) level groups but did not describe the reason why their pigs presented with such different endogenous OT levels. However, they did make the following interesting comment: “It is possible that animals presenting high PTOT [pretreatment OT levels] perceived more stress, which could influence outcome” [43]. This statement is counterintuitive: why should high endogenous OT levels lead to higher stress perception? The contrary would be expected since OT has anxiolytic effects. That being said, it is noteworthy that this “influence” may be reflective of an acute perception of threat and not a chronic disease state. Nevertheless, it does appear that the high levels of OT, coupled with the exogenous OT administration, are associated with a reduction of the cardiac OTR levels, either through desensitization or internalization [94]. This attenuation of the OTR expression may represent an adaptive response in that the exogenous infusion in this arm proved to be detrimental. Thus, the potential of OT to exert its cardio-protective effects seems to be at least in part dependent on the presence and levels of both its receptor and ligand. In fact, the protective effects of OT are mostly mediated though the OTR, both in response to normal adaptive stressors or to trauma and injury [84,95].

There are only a couple of reports which have actually looked at the interaction of the H_2_S and OT system directly, and they are discussed below. In an acute-on-chronic disease murine model of traumatic injury, the interaction of the H_2_S and OT system was shown in response to cardiovascular injury. Trauma significantly reduced cardiac OTR expression, and this downregulation was further aggravated in mice with genetic CSE deletion. In addition, the loss of cardiac OTR was restored by exogenous H_2_S administration through the slow releasing H_2_S donor morpholin-4-ium 4-methoxyphenyl(morpholino) phosphinodithioate) GYY4137 (GYY) [5,9]. Naive CSE knock out (ko) mice had lower levels of OTR [12], and similarly, naïve mice with a genetic deletion of OTR presented with a reduction of CSE expression [8]. Global genetic deletion of 3MST was shown to result in hypertension and cardiac hypertrophy in old age and was accompanied with increased anxiety-like behaviors [96]. Recently, in mice with a genetic mutation of 3MST (ΔMST), cardiac CSE and OTR was reduced both in the naive and post-injury state. Moreover, the mitochondrial complex IV activity was reduced in the ΔMST mice in comparison to the wild type mice after injury (see Table 1) [5].

## 5. Developmental Origins of Health and Disease: Heart

ELS is multifactorial, and a diversity of environmental influences can affect cardiovascular programming and the development of CVD in later life. Amongst these environmental influences, ELS plays a significant role, stemming from the womb, and includes maternal nutrition, smoking, alcoholism, medication/drugs, and illness [20] leading to vulnerability of the fetal cardiovascular system, e.g., morphological and functional adaptations that stiffen the vascular tree, small coronary arteries, endothelial dysfunction, reduced number of cardiomyocytes, atherogenic blood lipid profiles, and coagulopathies [20,97]. That these effects may stem from the womb was only discovered fairly recently due to pioneering work such as that by Higgins et al. [98] which linked pre-eclampsia with the high blood pressure of the offspring, which worsened as the offspring matured, as well as their keen suggestion that the prenatal environment was responsible rather than genetic defects. This was followed by the work of Barker and Osmond [99] that showed the association of infant mortality, childhood nutrition, and ischemic heart disease. More groundbreaking studies have subsequently led to the concept and active pursuit of a better understanding of how afflictions in utero influence later life, now called the developmental origins of health and disease (DOHaD) [100]. Recent evidence suggests that the cardiovascular programming leading to CVD disease, although the causes are varied, have common mechanisms. Albeit they are not fully delineated or understood, experimental modeling has implicated oxidative stress, NO, renin angiotensin system, nutrient-sensing signals, and gut microbiota dysbiosis [20,97,101,102]. As pointed out above, both the H_2_S and OT systems have been shown to interact in these very mechanisms instrumental in cardiac programming. 

The traditional approach to help prevent CVD focused on modifying behavioral patterns in adults. Recently, the American Heart Association has identified childhood as an important period to intervene for reducing the risk over the life span [2]. It is suggested that interventions reducing early risk factors may be more instrumental than interventions that attempt at remediating CVD later in life [3].

**Figure 1:**
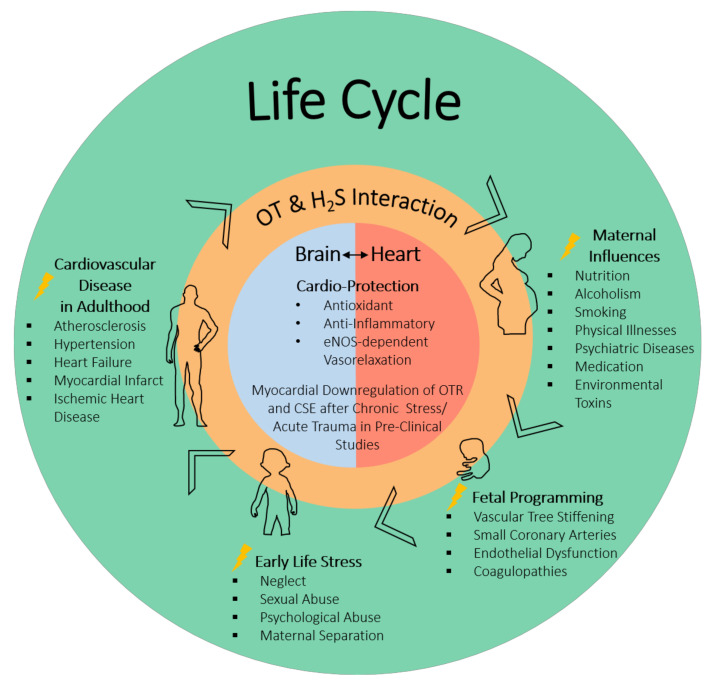
Interaction of OT and H_2_S in the brain and the heart: contribution to physical manifestation/protection of ELS-induced CVD in adulthood. ELS affects cardio-vascular programming and the development of CVD in adulthood. Pregnant women who experienced ELS may pass on ELS-triggered behavior/physical conditions: nutrition, smoking, alcoholism, medication/drugs, and illness. Long-term effects on the cardiovascular system of the fetus can be morphological and functional adaptations, stiffening of the vascular tree, small coronary arteries, endothelial dysfunction, reduced number of cardiomyocytes, atherogenic blood lipid profiles, and coagulopathies. Bringing together literature reports on OT and H_2_S suggests that both are significant common mediators of cardio-protective effects during development as well as in adulthood through their antioxidant and anti-inflammatory effects and eNOS-dependent vasorelaxation. Pre-clinical studies suggest that chronic stress and/or acute trauma leads to a downregulation of the OTR and CSE (the main endogenous H_2_S-producing enzyme in the vasculature). OT: oxytocin; H_2_S: hydrogen sulfide; ELS: early life stress; CVD: cardiovascular disease; eNOS: endothelial nitric oxide synthase; CSE: cystathionine-γ-lyase.

It is well established that psychological stress, e.g., ELS, is a known risk factor for the development and progression of CVD (see Figure 1) [59,103,104,105,106,107]. The OT/OTR system has been studied with regards to how it may impact on maternal behavior, optimism, and social reward perception, as well as anxiolytic effects and stress-related responses [65,108,109]. Psychological stress increases blood pressure and heart rate; the chemical blockade of the OTR was shown to worsen the cardiovascular response to stress [52,53,110,111]. In rodent models of maternal separation, the neonates respond with increased inflammation, and OT infusion has been shown to be beneficial [44,84]. Looking at the role of cardiac OTR in adult mice exposed to ELS, Wigger et al. found that there were “dose” response differences with regards to the expression of OTR and CSE, the most important H_2_S-producing enzymes in the heart [8]. They reported that neonatal chronic psychological trauma during a short-term separation stress (STSS) paradigm had the opposite effect on the expression of OTR in the heart in comparison to long-term separation stress (LTSS) (see Table 1) [8]. In these experiments on a psychological trauma model, “chronically” LTSS leads to a long-term reduction in cardiac OTR and CSE expression. The findings are in line with those reported by Merz et al. [9] in a combined acute-on-chronic physical trauma model, where there was a downregulation of the OTR in cardiac tissue. Interestingly, the authors speculate that the upregulation of the OTR in the heart in the STSS group may be mediating stress resilience, whereas the attenuated expression in the LTSS group mirrors stress-induced vulnerability [8]. It is noteworthy that in the ΔMST phenotype, resulting from a genetic mutation of 3MST, the naïve mice display a similar loss of cardiac CSE and OTR as reported for the chronic stress group and as a result of physical trauma [5]. This suggests a possible important, yet not fully resolved, role for 3MST in the context of stress-induced cardiovascular disease and further supports the mutual and interrelated roles of the H_2_S and OT systems in both physical and psychological trauma (see Table 1) [5].

Recent reports on the role of H_2_S in psychological trauma, in particular ELS, suggest an amelioration of colon stress related injuries by exogenous H_2_S administration [54]. Interestingly, OT administration, in this context, has also revealed colon-protective actions through anti-oxidative and anti-inflammatory properties [45]. The significant role of H_2_S in the prevention of ELS-driven development of adult CVD has been recently discussed by Hsu et al. [20,22,112]. H_2_S has shown beneficial results in ELS models of developmental hypertension: suramin-induced preeclampsia [46] and perinatal high fat diets [20,22,47], as well as improved depressive behavior induced by chronic unpredictable mild stress (see Table 1) [55].

## 6. Gender

Evidence from epidemiological studies has not only confirmed that ELS increases the incidence of CVD and blood pressure (inverse relationship between birth weight and blood pressure) in adulthood, but that it does so in a gender-specific manner [113]. In general, men have higher blood pressure during early adulthood, whereas females are normotensive, but when exposed to ELS, succumb to increased blood pressure [113,114]. Li et al. found that women who have experienced physical abuse and/or emotional neglect had a significantly higher risk for ischemic heart disease and for CVD in general [115]. Albus et al. report that microvascular dysfunction and vasospasm are associated with exposure to stress in women [1]. Interestingly, aging also increases the risk of CVD in women, with menopause increasing the risk and reducing the sex difference [113,114,116]. In particular, the incidence of postmenopausal women presenting with broken heart syndrome (Takotsubo cardiomyopathy), 90%, is striking [1]. There is evidence that both the OT and H_2_S systems play gender-specific roles, and the production of OT has been shown to vary between males and females [117]. In hypertensive rats, OT administration reduced blood pressure in male but not in female rats [48]. In contrast to the results in hypertensive rats, in a human study in healthy male subjects receiving intranasal OT led to an increase in blood pressure (see Table 2) [57].

Interestingly, in a psychological stress study in children, only prepubescent girls who had ELS with a history of physical abuse showed increased urinary levels of OT in response to a social stressor, while no differences were seen in boys with or without ELS [118]. Furthermore, the authors state that the fact that OT levels were much higher at baseline in the ELS girls suggests that this may reflect fundamental changes in the OT system after ELS in girls [118]. The possibility that these changes are protective or an indication of resilience is not addressed, but it is noteworthy that the control girls, in response to a social stressor, displayed increased cortisol concentrations, whereas the ELS girls had lower levels of cortisol before, during, and after the stress test. Again, no differences were found in boys [118]. The cycles of menstruation are also reported to influence OT levels [119]. There are gender-specific responses to OT during physical or psychological challenges [120,121] after exogenous administration of OT [119,122,123,124,125,126,127,128] as well as a tendency towards “tend and befriend” by women and “fight or flight” by men [119,129]. Intriguingly, in a study designed to address the tend-and-befriend response to stress in women, intranasal OT administration resulted in reduced heart variability in study subjects receiving support, which manifested in lower cortisol levels after the TSST, but only in the women with ELS who received support from a friend [130]. This finding is in contrast to the majority of the literature suggesting that oxytocin administration is contraindicated for individuals with ELS [83,131]. The above begs the question of the significant psychological role played by social support in the context of OT administration and its therapeutic potential.

Inherently, women have been shown to have a greater antioxidant capacity in their brains and therefore have been reported to be more protected than males under stressful situations, e.g., perinatal boys were shown to be more vulnerable to OT exposure while in the womb and have a higher risk of developing autism [95,132]. There are reported differences in OT and OTR levels in males and females, which are also well documented in animal models [119,129,133,134,135,136]. OTR is known to be modulated by gonadal hormones [80], and, in addition, both endogenous and exogenous steroids (e.g., estrogen and testosterone) were shown to influence OT/R expression [84,121]. In animal models, the hormonal status of the experimental animals is influenced by age, weight, menstrual cycle (the estrus phase being associated with higher OTR binding [137], high OTR levels in the luteal phase and the menstruation phase, and low OTR mid-cycle), menopause [78], ovariectomization, and castration, also decreasing OTR [80]. Sex hormones have also been associated with their ability to influence the CV-mediated effects of H_2_S in rats: females had increased CSE activity and higher myocardial H_2_S levels than males [49], and estrogen was shown to increase myocardial CSE expression and endogenous H_2_S synthesis, which in turn, was concomitant to reduced inflammation and oxidative stress [138]. In contrast, H_2_S biosynthesis in the rat aorta was reported to be responsive to androgen hormones and not to estrogen or progesterone [139]. In humans, gender specific differences in H_2_S bioavailability were recently reported between Caucasian males and females, leading the authors to conclude that H_2_S bioavailability could be considered a biomarker for CVD in a gender specific manner (see Table 2) [58].

## 7. Brain Heart Connection

When looking at the brain and nervous system there is also evidence for a bi-directional effect of the H_2_S and OT systems in the interaction of the CV and CNS. Particularly of interest to the relationship of the H_2_S and OT systems in the regulation of fluid homeostasis are the supraoptic (SON) and paraventricular nuclei (PVN) [14] of the hypothalamus. The central integrative structure for the maintenance of osmolality, blood, and body fluid volume is the hypothalamus [140], which regulates blood pressure and heart rate in response to changes in peripheral fluid homeostasis [12,14]. In experimental models, dehydration (48 h) led to a significant increase in plasma OT and OT mRNA in the PVN [141]. Furthermore, chronic selective activation of PVN neurons producing OT attenuated myocardial injury and reduced mortality, concomitantly increasing cardiac parasympathetic tone [51,142]. H_2_S, suggestive of its role in the regulation of autonomic and endocrine functions, was shown, in a dose-dependent manner, to depolarize magnocellular neurons of the PVN [12,143]. The intra-cerebro-ventricular administration of an H_2_S releasing salt (sodium sulfide (Na_2_S)) in water-deprived (24 h) rats increased plasma levels of OT, while decreasing hypothalamic nitrate/nitrite [14].

Recently, in a porcine model of acute subdural hematoma (ASDH), the localization of OT/R was confirmed in the hypothalamus and was also found to co-localize with CSE [7,12]. Interestingly, CSE and OTR displayed reciprocal expression patterns in the cerebellum, suggesting a more complex relationship and that different brain regions may differ in the interaction of the OT/H_2_S systems [12]. Furthermore, the authors observed the activation of the H_2_S and OT systems in the prefrontal cortex, which may assume particular relevance, because this is one of the brain regions reported to be dysregulated in posttraumatic stress syndrome (PTSD): the presence of these two systems may be indicating potentially relevant biological mechanisms of ASDH-induced PTSD [6,144,145].

## 8. Vagus Nerve—H_2_S and Oxytocin

**Figure 2:**
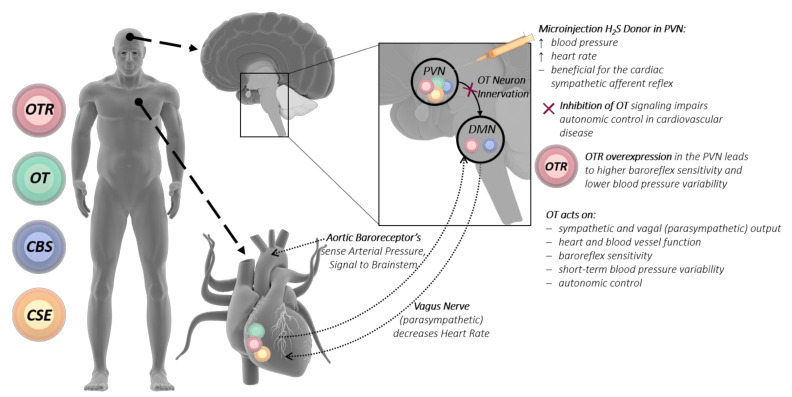
H_2_S and OT directly influence the vagus nerve, connecting heart and brain. Baroreceptors in large arteries sense the arterial pressure and signal to the DMN of the brainstem. The DMN also receives input from OT neurons of the hypothalamic PVN, which centrally regulates cardiovascular homeostasis. The vagus nerve (parasympathetic) signals from the DMN to the heart and decreases the heart rate. Notably, the OTR, OT, CBS, and CSE are expressed within the PVN. The OTR and CBS are expressed in the DMN, while cardiac tissue expresses the OTR, OT, and CSE. These expression patterns suggest a mutual interplay of the OT and H_2_S systems, connecting the heart and the brain. Pre-clinical studies show that acute trauma and/or chronic stress affects cardiac endogenous OT/R and CSE levels, thereby affecting physiological responses that influence the development of CVD. DMN: dorsal motor nucleus of the vagus nerve; PVN: paraventricular nucleus; OT: oxytocin; OTR: oxytocin receptor; CSE: cystathionine-γ-lyase; CBS: cytathionine-β-synthase; CVD: cardiovascular disease. Illustrations of the human, heart and brain were taken from the Library of Science and Medical lllustrations (somersault18:24, https://creativecommons.org/licenses/by-nc-sa/4.0/).

A proposed mechanism for the interaction of the H_2_S and OT systems between the brain and heart is the vagus nerve. The vagus nerve is a very important player of the autonomic nervous system, regulating metabolic homeostasis and connecting the brain with the heart. This is mediated by afferent vagal nerve fibers (80%), which control sensory signals towards the brain, and efferent vagal nerve fibers (20%), which conduct signals towards peripheral organs such as the heart, lungs, and gastrointestinal tract. Within the brain, the nerve fibers of the vagus nerve terminate in the nucleus tractus solitaries, which is among others connected to the amygdala, the hypothalamus, and the orbitofrontal cortex [146]. Under stress conditions, the vagus nerve is an important player in keeping the heart from being overstimulated by the sympathetic nervous system [147]. Oxytocinergic cells from the PVN are directly connected to the vagal nuclear complex, where OT acts on sympathetic and vagal output to control heart and blood vessel function [50] (see Figure 2). The inhibition of OT signaling to the vagus nerve could lead to impaired autonomic control in cardiovascular disease [50,146]. OTR in the PVN fine-tunes the tonic neural control of baroreflex sensitivity, short-term blood pressure variability and autonomic control in cardiovascular diseases [50].

The OT-producing magnocellular neurons of the PVN have been shown to directly excite cardiac vagal neurons [51], and the OT system has been implicated in the maintenance of cardiovascular homeostasis and parasympathetic cardiac activity (especially in stress and anxiety). OTR-overexpressing (only in PVN) rats have higher baroreflex sensitivity and lower blood pressure variability than controls [50]. The chronic stimulation of OT-producing neurons in the PVN, activating cardiac vagal neurons, increased the parasympathetic tone and reduced cardiac hypertrophy [51]. In myocardial ischemia/reperfusion (I/R) injury, activation of the vagus nerve, attenuated severe arrhythmias, led to a reduction of free radical blood levels and reduced mortality [148]. The stimulation of the vagal dorsal motor nucleus in the brainstem lowered respiratory frequency and induced bradycardic responses [149]. Both specific activations of OT-producing neurons in the PVN and subcutaneous OT administration revealed specific parasympathetic effects [50,150]. Conversely, an intracerebral injection of OT at basal state and in response to stress (air jet) had no effect on cardiac function, whereas intracerebral injection of an OT antagonist worsened the cardiovascular response to stress [53].

Vagus nerve stimulation, in a porcine sepsis model, reduced cellular myocardial depression and cardiac mitochondrial dysfunction [151]. OT also mediated cardio-protection through the cardiovascular, respiratory, and immune response, thus strengthening an autonomic cholinergic link [150]. The authors could show in endotoxemic rats that OT administration (subcutaneously) reduced tachypnea and was beneficial for cardiovascular-respiratory coupling, as assessed by the spectral components of heart rate variability [150]. Heart rate variability is the main read out for vagus nerve activity [152]. Vagus nerve stimulation improved hypotension, while reducing tumor necrosis factor and in the end prolonged survival [152,153]. Studies in humans implicate low vagus nerve activity to increased insulin resistance and atherosclerosis [154,155,156,157,158], whereas increased vagus nerve activity is reported to reduce atherosclerosis [159,160,161,162,163,164,165]. An interesting observation is that OT may be also mediating vagus nerve stimulation by the probiotic bacteria *L. reuteri* [166], which has been shown to stimulate OT production in the PVN. 

The literature on the role of H_2_S in vagal nerve-mediated cardiovascular function is scarce at best, but there is a report that microinjection of an exogenous H_2_S donor (NaHS) into the dorsal motor nucleus of the vagus nerve elicited significantly decreased respiratory frequency and heart rate [167]. In a model of chronic heart failure, microinjection of GYY into the PVN led to higher renal sympathetic nerve activity, increased blood pressure, and heart rate and was beneficial for the cardiac sympathetic afferent reflex [168], whereas hypothalamic H_2_S administration led to reduced blood pressure [32]. The endogenous H_2_S-producing enzyme CBS has been localized in the dorsal motor nucleus of the vagus nerve, suggesting local H_2_S production [164,166]. Incidentally, OTR binding and gene expression were also localized in the dorsal motor nucleus [169] (see Figure 2). H_2_S is also reported to influence homeostatic processes and neuronal excitability regulating neurotransmission, adjusting the osmotic-induced neurohormone release such as OT [14,143,170,171]. “H_2_S-dependent oxygen sensing” is CSE-mediated in the carotid body by glomus cells, its inhibition leads to failure of the hypoxic response accompanied by a loss of catecholamine release [172,173]. Taken together, the results reported above demonstrate that both H_2_S and OT directly influence the vagal nerve through stimulation of the PVN, which leads to affecting the heart and the cardiovascular system: blood pressure, heart rate, heart rate variability, and cardiovascular tone (see Figure 2). OT in concert with H_2_S could have a direct physiological interaction, affecting the baroreceptor sensitivity and reaction to hypoxic events in cardiovascular stress.

## 9. Conclusions

The evidence that ELS leads to cardiovascular programming that manifests in CVD is incontrovertible, but the mechanisms by which this occurs are not fully understood. In this perspective review, the gasotransmitter hydrogen sulfide (H_2_S) and the neuroendocrine oxytocin (OT) systems were shown to interact and play parallel roles in the heart and brain in response to trauma, and evidence was provided to support their potential role as mediators in the ELS developmental origins of CVD. It is noteworthy that H_2_S and OT/R share: (i) signaling cascades that converge on the same signaling pathway, anti-inflammatory antioxidant properties, (ii) reduce atherosclerosis, and (iii) are cardio-protective in both physical and psychological trauma models. Furthermore, evidence was put forward supporting the role of the vagus nerve as a putative link for the interaction of the H_2_S and OT systems between the brain and the heart. The dysfunction of either system is associated with increased risk of hypertension and CVD. Nonetheless, discordant findings regarding the varied effects of OT exposure in early life and the varied responses to OT administration in individuals with ELS suggest that there is a need to better understand the discrepancy between the circulating levels of OT and the OTR tissue expression levels. This implies that multiple factors may be at play in the regulation of the OT system, e.g., stress affecting endogenous receptor ligand levels, ultimately affecting physiological response. In other words, the potential of OT to exert its cardio-protective effects seems to be at least in part dependent on the presence and levels of both its receptor and ligand. The fact that the loss of cardiac OTR expression could be restored by exogenous H_2_S administration might be an alternative to direct OT administration, in potentiating the OT system through collateral support, which may lead to OTR upregulation and restore signaling. The protective effects of OT are mediated through the OTR in response to normal adaptive stressors to trauma and injury (acute and/or chronic). The evidence that both the OT and H_2_S systems display gender specific roles, especially in response to ELS, mandates the need to include both sexes in experimental designs to fully comprehend the manifold interactions. The complexity of these findings warrants further study in clinically relevant animal models of ELS. These clinical models should include large animals (small rodent models are notorious for manifesting robust responses, which do not translate to the clinic) of both sexes, with comorbidities resembling those in the targeted patient populations. Thus, a better understanding of the complex interaction of the OT and H_2_S systems in ELS-mediated CVD may provide an opportunity to decipher the mutual interplay of the body and mind in CV health and disease.

## Figures and Tables

**Table 1:** Summary of animal studies: effects of stress/trauma/comorbidity and treatment on the cardiovascular system, H_2_S system and the OT/OTR system.

Author and Year	Species	Type of Stress/Trauma/Comorbidity/Treatment	Read Out (Cardiovascular/H_2_S/OT/OTR)
**Physical**			
Trautwein et al., 2021 [5]	Mice	naïve ΔMST animals Hemorrhagic Shock wt Hemorrhagic Shock & Blunt Chest Trauma wt	Constitutive CSE & OTR in cardiomyocytes CSE & OTR↓ CSE & OTR↓ CSE &OTR↓↓
Merz et al., 2020 & Nußbaum et al., 2016 [10,31]	Swine (hypercholesteremic)	Septic Shock (vs. sham animals)	Systemic Troponin↑ ↓ cardiac output CSE & OTR↓
Merz et al., 2018 [9]	Mice CSE^−/−^ (vs. wt)	native wt Blunt Chest Trauma (& cigarette smoke exposure (CS)) Blunt Chest Trauma CSE^−/−^ (& CS) Blunt Chest Trauma CSE^−/−^& GYY4137 administration (& CS)	Constitutive OTR in cardiomyocytes OTR↓ OTR↓↓ OTR↑↑
Meng et al., 2015 [24]	Rats	Physical Myocardial Ischemia/Reperfusion Injury GYY4137 administration	Myocardial Ischemia/Reperfusion led to: ↑CSE mRNA expression in the myocardium ↓CSE activity in the myocardium GYY4137 administration led to: ↓CSE mRNA expression in the myocardium ↑CSE activity in the myocardium ↑cardiac ejection fraction ↑fractional shortening ↓ischemia area alleviated histological injury ↓oxidative stress ↓apoptosis
Merz et al., 2017 [30]	Swine (hypercholesteremic vs. young german)	Septic Shock (vs. sham animals)	Acute circulatory failure Coronary Arteries: ↓CSE in the media No CBS in the media; but localized to the adventitia and atheromatous plaques No 3MST
Mani et al., 2013 [41]	Mice CSE^−/−^ (vs. wt)	Knock out & atherogenic diet NaHS administration	Early fatty streak lesions in the aortic root ↑Plasma levels of cholesterol & low-density lipoprotein cholesterol Hyperhomocysteinemia ↑ Lesional oxidative stress and adhesion molecule expression ↑Aortic intimal proliferation CSE^−/−^ treated with NaHS: inhibited the accelerated atherosclerosis development
Kobayashi et al., 2009 [42]	Rabbit	Myocardial Ischemia/Reperfusion Injury	Postinfarct treatment with OT led to: ↑Left ventricular function & remodeling ↓Infarct size OTR↑
Authier et al., 2010 [43]	Swine	Myocardial Infarct	Swine treated with OT immediately after the myocardical infarct for up to seven days: ↓Fraction shortening & no effect on lesion size 8d post myocardial infarct: swine with ↑basal endogenous OT levels receiving OT treatment: ↓Ventricular function & ↑infarct size 28 d post myocardial infarct: in comparison to placebo animals with ↑endogenous OT levels, swine with ↓endogenous OT: ↓infarct size OT administration led to: ↓Cardiac OTR in ↑endogenous OT animals, but not in ↓endogenous OT animals
Klein et al., 2018 [44]	Rats	Nutrient insufficiency in neonates	OTR-rich brain regions show: NF-kB was retained ↑ in the cortex, striatum nuclei, and medial preoptic nucleus NF-kB was ↓ & unchanged in nucleus of the solitary tract, paraventricular nucleus, and supra-optic nucleus Unprimed by colostrum: ↑Endoplasmic reticulum stress in solitary tract Primed by colostrum: ↓Endoplasmic reticulum stress in solitary tract
Iseri et al., 2005 [45]	Rats	Sepsis (vs. sham animals)	Sepsis led to: ↑Malondialdehyde (indicating lipid peroxidation in colon, uterine & liver ↓Glutathione (key antioxidant) in colon & uterine ↑Myeloperoxidase (indicating neutrophil infiltration) in colon & liver ↑Collagen levels in the uterus & liver ↑serum TNF-α levels Subcutaneous OT treatment reversed the above negative effects induced by sepsis, while hepatic glutathione levels were not affected
Tain et al., 2016 [46]	Rats	Pregnant maternal suramin treatment	Induced programmed hypertension in male offspring ↑Plasma nitric oxide synthase inhibitor (ADMA) Maternal n-acetylcysteine administration prevented hypertension Protective effects of n-acetylcysteine: ↑Plasma glutathione level, ↑3MST, & restoration of suramin-induced reduction in H_2_S synthesis in the kidneys
Tai et al., 2016 [47]	Rats	High-fat diet from weaning on/prenatal dexamethasone	Prenatal dexametahsone and postnatal high-fat diet induced programmed hypertension in adult offspring Prevented by maternal n-acetylcysteine therapy ↑gene expression of H_2_S-generating enzymes ↑Renal 3MST protein levels and activity ↑Plasma glutathione level, ↓oxidative stress
Petersson et al., 1997 [48]	Rats	Spontaneously hypertensive, subcutaneous OT or saline for 5 days to ♂ and ♀ rats	♂: ↓blood pressure, no effect on heart rate, (vs. saline-treated controls), effect was gone 3d after the last injection ♀: no effect on blood pressure and heart rate
Melnik et al., 2017 [49]	Rats	Castrated	Castration in ♂ led to:↓CSE, ↓H_2_S, ↑proliferation, polyploidization & apoptosis in myocardium (vs. ♀) Castration in ♀ led to: ↑CSE, ↑H_2_S, ↓proliferation, polyploidization & apoptosis in myocardium (vs. ♂)
Lozic et al., 2014 [50]	Rats	Air-jet (overesxpressing OTR in paraventricular nucleus vs. sham) Pretreatment of OTR overexpressing rats with OT	At baseline conditions: rats overexpressing OTR: ↑baroreceptor reflex sensitivity, ↓blood pressure variability (vs. sham) Exposure to stress: ↑blood pressure, blood pressure variability & heart rate in all rats Sham animals: ↓baroreceptor reflex sensitivity during stress Pretreatment of OTR overexpressing rats with OT: ↓baroreceptor reflex sensitivity, ↑blood pressure and heart rate variability (baseline and stress) Pretreatment of sham rats with OT: ↓baroreceptor reflex sensitivity, ↑blood pressure variability (baseline and stress) only ↑ heart rate variability during stress
Garrott et al., 2015 [51]	Rats	Left ventricular hypertrophy, heart failure, OT treatment	Activation of hypothalamic OXT neurons to elevate parasympathetic tone let to: ↓cellular hypertrophy, IL-1β & fibrosis with OT treatment: Cardiac contractility parameters were significantly ↑ Heart rate sensitivity to β-adrenergic stimulation was ↑
**Psychological**			
Wigger et al., 2020 [8]	Mice	Maternal Separation (Early Life Stress) LTSS (long) STSS (short)	CSE & OTR↓↓ CSE↓ & OTR↑↑
Peters et al., 2014 [52]	Mice	Chronic psychosocial stress: chronic subordinate colony housing Infusion of OT (intracerebroventricular for 15 days) high and low dose	High dose OT led to: anxiogenic phenotype, OTR binding in septum, amygdala & median raphe nucleus low dose OT led to: prevents hyper-anxiety, thymus atrophy, adrenal hypertrophy & ↓adrenal ACTH sensitivity (in vitro)
Wsol et al., 2008 [53]	Rats	Alarming stressor (air jet) Animals received intracerebroventricular: vehicle, OT, or OT-antagonist	Under resting conditions: infusions no effect on cardiovascular parameters alarming stressor evoked: ↑mean arterial blood pressure ↑heart rate Animals that received the OT antagonist (vs. OT and vehicle treated): ↑↑mean arterial blood pressure ↑↑heart rate
Li et al., 2016 [54]	Mice	Maternal separation (vs. control without maternal separation)	Maternal separation led to changes in the proximal colon: ↓crypt lengths, ↓goblet cells per crypt, ↓glutathione peroxidase activity, ↑expression of thiobarbituric acid reactive substances & inducible nitric oxide synthase mRNA, ↑IL-6, TNFα & myeloperoxidase Administration of NaHS led to: ↓↓negative effects
Liu et al., 2017 [55]	Rats	Chronic unpredictable mild stress	↑Depressive-like behavior, ↑hippocampal endoplasmic reticulum stress & ↓Sirt-1 NaHS administration led to: ↓Depressive-like behaviors, ↓hippocampal endoplasmic reticulum stress & ↑Sirt-1

Abbreviations: H_2_S = hydrogen sulfide; OT = oxytocin; OTR = oxytocin receptor; CSE = cystathionine γ-lyase; 3MST = 3-mercaptopyruvate sulphurtransferase; ΔMST = genetic mutation of 3MST; CS = cigarette smoke exposure; CBS = cystathionine β-synthase; NaHS = Sodium hydrosulfide; wt = wild type; CSE^−/−^ = CSE knock out; ACTH: adrenocorticotropic hormone. ↓ slightly down, ↓↓ strongly down, ↑ slightly up, ↑↑ strongly up.

**Table 2:** Summary of human studies: effects of stress/trauma/comorbidity and treatment on the cardiovascular system, H_2_S system and OT/OTR system.

Author and Year	Species	Type of Stress/Trauma/Comorbidity/Treatment	Read Out (Cardiovascular/H_2_S/OT/OTR)
Sun et al., 2007 [35]	Human (Adult)	Ever-treated hypertensive patients (vs. control patients) Ever-treated hypertensive patients with grade 2 and 3 hypertension (vs. control patients)	↓Plasma H_2_S levels ↑Homocysteine ↓Plasma H_2_S levels
Chen et al., 2007 [36]	Human (Children)	Essential hypertension (vs. children with normal blood pressure)	↓Plasma H_2_S levels
Polhemus et al., 2014 [17]	Human (vs. age matched controls)	Heart failure	End stage cardiomyopathy, reduced heart function ↓Plasma H_2_S levels
Polhemus et al., 2015 [56]	Human (vs. healthy sibjects)	Heart failure Administration of SG1002	No changes in safety effects & plasma H_2_S levels in healthy subjects and heart failure patients
Meusel et al., 2021 [57]	Human (only males)	Intranasal OT (vs. placebo)	↑Resting muscle sympathetic nerve activity ↑Resting diastolic blood pressure No effect on: systolic and mean arterial blood pressure, heart rate, baroreflex sensitivity at vasoactive drug challenge, ACTH, cortisol or norepinephrine
Rajpal et al., 2018 [58]	Humans	Patients with/without cardiovascular disease	Caucasian ♀ with cardiovascular disease: ↓plasma acid labile sulfide levels (vs. ♀ without cardiovascular disease) Caucasian ♂ with cardiovascular disease: ↓plasma bound sulfane sulfur levels (vs. ♂ without cardiovascular disease) No gender H_2_S bioavailability differences in african americans, but: general ↓H_2_S bioavailability (vs. caucasians) ↑CSE 1364 G-T allele frequency in patients with cardiovascular disease (vs. without cardiovascular disease) Plasma H_2_S bioavailability was predictive for cardiovascular disease in caucasian subjects

Abbreviations: H_2_S = hydrogen sulfide; OT = oxytocin; CSE = cystathionine γ-lyase; ACTH: adrenocorticotropic hormone. ↓ slightly down, ↑ slightly up.
